# A Remotely Delivered Weight Management Service to Support Existing Obesity Services in the UK National Health Service: Preliminary Findings From an Early-Stage Service Evaluation

**DOI:** 10.2196/71914

**Published:** 2025-10-15

**Authors:** Giulia Spaltro, Michael Whitman, Rebecca Richards

**Affiliations:** 1 Second Nature Healthy Habits Ltd, 58 Wood Lane, London, W12 7RZ, United Kingdom, 44 020 46025555

**Keywords:** obesity, diabetes, weight loss, weight management, digital health, digital health intervention, multidisciplinary, behaviour change, disordered eating, dialectical behavioral therapy

## Abstract

**Background:**

Specialist weight management services (SWMSs) in the UK National Health Service (NHS) face long waiting lists and limited resources. Remotely delivered SWMSs may support existing NHS services to increase access to treatment for patients living with obesity; however, evidence of remotely delivered services working to support NHS SWMSs in practice remains limited.

**Objective:**

This study aims to explore the potential effectiveness, feasibility, and acceptability of Second Nature’s remotely delivered SWMS for adults living with obesity referred from existing NHS SWMSs. Preliminary findings from the first phase (Preparing for weight loss) of a 3-phase remotely delivered SWMS are presented.

**Methods:**

A total of 39 adults (age range 23‐74 years, mean age 45.6, SD 12.1; 74% female) completed a 16-week intervention, following referral from NHS SWMS leads. Eligible participants were assessed by a multidisciplinary team and allocated to one of three interventions: (1) a psychologically informed app-based intervention, (2) a Dialectical Behavioral Therapy (DBT)-based skills training group intervention, and (3) one-to-one psychological support. The primary outcomes were weight change (kg) and percentage weight change following completion of the intervention. Secondary outcomes included psychological distress, emotional eating, health-related quality of life, physical activity, emotion regulation, intervention feasibility, and acceptability.

**Results:**

At 16 weeks, the mean weight change was −2.2 kg (SD 5.16), or −1.6% of body weight. Participants in the app-based intervention lost the most weight (−2.8kg), and participants in the one-to-one psychological support intervention lost the least weight (−1.3kg). Psychological distress was reduced to below the clinical threshold (mean score 0.95, SD 0.62). Emotional eating behaviors and difficulties in emotion regulation also decreased (mean change scores −3.2, SD 6.4 and mean −11.6, SD 13.9, respectively). Health-related quality of life saw improvements in self-care, usual activities, and anxiety and depression, while participants’ challenges with mobility, and pain and discomfort remained unaffected. Subjective ratings of health status improved by 17.4%. There were no significant changes in physical activity levels, with most participants remaining “Inactive” or “Moderately inactive.” Engagement with intervention sessions was high (93.7%) and the attrition rate was 27.4%. Participants rated their satisfaction with the intervention at 9/10 and highlighted key benefits, including improved mental well-being, healthier habits, and supportive coach relationships. Suggested improvements included greater scheduling flexibility, enhanced app functionality, and more accessible physical activity support.

**Conclusions:**

This preliminary service evaluation suggests that a remotely delivered SWMS has the potential to be effective, feasible, and acceptable for NHS-referred patients in the United Kingdom. Changes observed across several key measures point to clinically significant benefits, reinforcing the potential of this approach. A full evaluation of all 3 phases of this service with a larger sample size is required to support these early findings.

## Introduction

### Background

Around 1 in 3 adults in the United Kingdom are affected by obesity [1-4] (defined as abnormal or excessive fat accumulation that presents a risk to health, with a BMI of ≥30 [1]). Many people living with obesity are also burdened by weight-related conditions such as cardiovascular disease, type 2 diabetes, liver disease, and sleep apnea and are at risk of many cancers [1,5]. In addition to poorer physical health, obesity is associated with poorer mental health [6-8] and lower quality of life [9]. Due to the wide-ranging negative impact on the health and well-being of people living with obesity, comprehensive treatment that considers the biological, psychological, and social determinants of obesity is imperative. This approach aligns with recent guidance from an international expert working obesity group which suggested the outcomes of treatment for clinical obesity should focus on easing the symptoms of obesity, rather than focusing on weight loss [10].

Individuals with a BMI ≥30 and weight-related comorbidities are eligible for Specialist Weight Management Services (SWMS) in the UK National Health Service (NHS) [11], where specialist assessment, monitoring, and comprehensive tailored treatment by a physician-led multidisciplinary team (MDT) is provided [12-14]. An MDT typically includes a physician, nurse, dietitian, psychologist, and a physiotherapist or exercise therapist, each with a specialist interest in obesity. Treatment in SWMSs is typically tailored to the patient’s presentation but can involve support to make dietary changes, increase physical activity, and psychological support to overcome psychological barriers to weight loss, as well as pharmacotherapy where required or accessible [15]. However, due to the comprehensive and tailored treatment offered, SWMSs are associated with a high financial cost [16]. In addition, there appears to be wide variation in treatment protocols and MDT structures within and between SWMSs, which may be due to funding limitations [16]. Nevertheless, research suggests that SWMSs in the United Kingdom can be an effective obesity treatment [17,18], though evidence is still limited [19,20]. For example, a systematic review of 19 studies of SWMSs in the United Kingdom reported positive effects on weight (specifically, 43.4%% and 29.4% achieved ≥5% and 10% weight loss, respectively), BMI, glycemic control, blood pressure, and physical activity at 12 months as a result of the comprehensive and tailored treatment offered [17].

Due to the high cost associated with traditional in-person SWMS and funding limitations, the provision of, and access to, SWMSs across the United Kingdom remains limited and varies geographically [18,19,21,22], and treatment can last anywhere between 4 and 24 months [17]. Furthermore, services face long waiting lists, as well as a lack of treatment continuity and flexibility [17,21]. Overall, these barriers can result in suboptimal protocols, treatment delays, and ultimately, adversely affect patient outcomes [17,22,23].

Digital weight management interventions (DWMIs) are promising solutions for addressing gaps and issues in the current service provision of traditional SWMSs that historically have been provided in person [18,24-26]. Increased access to services, increased convenience, more frequent care, resource- and cost-savings, and scalability potential are among the benefits of DWMIs to support people living with obesity and related conditions [18,27,28]. Previous studies, including 3 systematic reviews, have shown that DWMIs can be as effective as in-person interventions for weight loss and related outcomes for people with obesity [26,29-32], including within the context of SWMS [18,33,34]. For example, preliminary findings from a service evaluation of a remotely delivered, semaglutide-supported SWMS for adults living with obesity further suggested DWMIs have the potential to be effective, feasible, and acceptable for self-paying consumer adults with obesity in the United Kingdom [35]. In addition, a dietetic weight loss app-based program was found to be as effective and feasible when delivered remotely from a hospital-based SWMS to their usual face-to-face care [36]. Furthermore, a recent retrospective analysis of a 12-month app-based DWMI for 1130 patients living with obesity (with or without type 2 diabetes), referred from NHS primary care services, showed a statistically significant mean percentage weight loss of 9.1% (SD 9.6%), at 1 year and 11.8% (SD 10.9%), at 2 years [28].

Despite these early findings, real-world evidence of the potential for DWMIs to support SWMS in the UK NHS remains limited [37]. Further research is still needed to assess the potential of a remotely delivered SWMS, which includes assessment and triaging to the appropriate interventions, as well as integration with the referring clinical teams in the NHS.

### This Study

To build on the growing real-world evidence base, this study explored the potential of Second Nature’s remotely delivered pilot SWMS to support existing services in the UK NHS to increase access to treatment for people living with obesity. This pilot service evaluation reports on the preliminary findings of the potential effectiveness, feasibility, and acceptability of the first intervention phase of a 3-phase remotely delivered SWMS for patients living with obesity referred from NHS SWMSs.

## Methods

### Overview

This manuscript was prepared in accordance with the SQUIRE 2.0 (Standards for Quality Improvement Reporting Excellence, Version 2) guidelines [38]. A completed checklist is included in Checklist 1 to support comprehensive reporting of this service evaluation.

This service evaluation used a retrospective analysis of preliminary data from participants referred to Second Nature Ltd by the NHS. The study only involved the use of previously collected, anonymized data that could not be traced back to identifiable individuals. As part of their referral, participants were required to provide informed consent for their anonymized data to be collected for research purposes, including analysis and publication. A privacy policy detailing data collection, storage, and processing is hosted on the Second Nature website and participants were invited to provide informed consent to the policy upon enrollment in the intervention [39].

All collected data were stored on a cloud-based encrypted storage service and accessed via company-owned password-protected devices. For the purpose of this evaluation, participant data were deidentified and pseudonymized using identification numbers. As per the General Data Protection Regulations (GDPR), participants could request to have their information deleted at any time [40].

### Participants

Participants included in this analysis were screened and referred via secure NHS email to Second Nature by their local NHS SWMS lead for weight management support. Participants were referred to Second Nature if they were deemed clinically suitable for the service by the referrer, in line with our inclusion and exclusion criteria (Textbox 1). A second screening process was conducted by a patient coordinator at Second Nature to confirm the eligibility of referrals.

Textbox 1. **Service inclusion and exclusion criteria.**

**Inclusion criteria**

- Over 18 years old
- BMI ≥35 in the presence of diabetes or other weight-related conditions (lowered to BMI ≥30 if screened and referred specifically by NHS SWMS service leads)
- Or BMI ≥40 without weight-related conditions (reduced by 2.5 kg/m² of BMI in Black African, African Caribbean, and Asian groups)
- The underlying causes of overweight or obesity need to be assessed
- The person has complex disease states or needs that cannot be managed adequately in lower-intensity weight management interventions
- Conventional treatment has been unsuccessful
- Specialist interventions (such as a very-low-calorie diet) may be needed
- Drug treatment is being considered for a person with a BMI ≥50 kg/m²
- Bariatric surgery is being considered
- Access to a digital device and comfortable using technology

**Exclusion criteria**

- Current or planned pregnancy
- An unstable condition that does not warrant weight management at present
- Recent complicated myocardial infarction or stroke, or awaiting further investigation
- Blood pressure at rest above 180 mm Hg systolic, 120 mm Hg diastolic
- Alcohol or drug misuse
- Diagnosed, active eating disorder or presenting with active purging behaviors (eg, laxative misuse, self-induced vomiting in the absence of a diagnosed eating disorder)
- Undergoing active cancer treatment
- Acute infection
- Unstable major psychiatric illness
- Recent bariatric surgery (within the last 2 years)

### Intervention Description

Second Nature’s remotely delivered SWMS consisted of three phases: (1) “Preparing for weight loss” (16 weeks), (2) “Active weight loss” (12 weeks), and (3) “Weight loss maintenance” (24 weeks). Participants were assessed at baseline and at completion of each of the subsequent intervention phases. In this study, we report outcomes for the first 39 participants who completed phase 1 (“Preparing for weight loss”). A further 6 participants took part in the intervention; however, they were not included in this evaluation as they had not yet completed it at the time of analysis.

The aim of phase 1 was to prepare participants for a future behavioral weight loss intervention (phase 2) by reducing psychological barriers to making changes to their diet and lifestyle, including psychological distress, emotional eating, and disordered eating behaviors [41-45].

Participants were triaged to one of 3 interventions based on a comprehensive assessment process which included validated questionnaires and a 50-minute video consultation with a health care practitioner (either a practitioner psychologist or health coach). Ahead of each initial consultation, participants with a risk score >0 (CORE-OM) were contacted by the safeguarding team for a risk assessment to ensure patient safety and suitability for the intervention.

Participants with a mean psychological distress score ≥1 were allocated to a practitioner psychologist for their initial video consultation, while participants with a score <1 had their initial consultation with a health coach. This decision was informed by the scoring guidelines of the scale selected for measuring psychological distress (CORE-OM).

Intervention allocation was informed by initial assessment outcomes and subsequently reviewed by a MDT. The MDT consisted of a physician, 2 practitioner psychologists, a dietitian and exercise specialist, 2 nutritionists (supervised by a dietitian), and a patient coordinator.

Interventions included: (1) a psychologically informed app-based intervention, (2) a Dialectical Behavioral Therapy (DBT)-based skills training group intervention, and (3) one-to-one psychological support. Each intervention was delivered remotely and lasted 16 weeks.

#### Psychologically Informed App-Based Intervention

Participants considered to have mild to moderate emotional eating or weight-related psychological distress were allocated to this intervention. The intervention consisted of personalized and modularized app-based content covering topics such as nutrition for weight loss, the psychology of eating, challenging all-or-nothing thinking, the science of habits, and mindful eating [41,42,46]. Module content was informed by the COM-B model of behavior change and the Behaviour Change Wheel [47]. This intervention included behavior change techniques [48] shown in previous studies to be associated with improved outcomes in diet and physical activity and recommended by the National Institute for Health and Care Excellence (NICE) guidelines for individual approaches to behavior change [49].

Participants were supported through the modules with 8 fortnightly video calls (30‐40 minutes) with their assigned health coach (registered or associate nutritionist, supervised by a registered dietitian). The assigned health coach tailored the order of modules to each participant’s presentation. On weeks where there were no calls, the health coach conducted a structured in-app text-based check-in using a one-to-one private chat feature. Participants were guided to reflect on the content of the educational modules, explore barriers to change, set goals, practice new skills, and develop a personalized plan to support realistic and sustainable lifestyle changes.

#### DBT-Based Skills Training Group Intervention

Participants who were considered to have moderate to high levels of emotional eating, or moderate to high levels of weight-related psychological distress, or symptoms of binge eating, were allocated to this intervention. The aim of this intervention was to reduce difficulties with emotion regulation, emotional eating, and disordered eating behaviors and was informed by Safer et al’s manual [50]. DBT, originally developed to treat borderline personality disorder [51], has been shown to be an effective treatment for binge eating disorder [52-55], at comparable levels of clinically meaningful change in global eating disorder psychopathology to cognitive behavioral therapy (CBT) [53,56]. Furthermore, there is preliminary evidence to suggest that DBT-based skills training groups can decrease binge eating severity and emotion regulation difficulties in individuals living with obesity without binge eating disorder [57,58].

In this intervention, participants were supported to build awareness of, and practical experience in, the 4 pillars of DBT: mindfulness, emotion regulation, distress tolerance, and interpersonal effectiveness, through weekly group video sessions (1.5 hours each) via Zoom (Zoom Communications Inc) led by a practitioner psychologist. Following each group session, participants were emailed a copy of the educational materials used during the sessions and were provided with digital access to a worksheet to complete before the next group session. Participants were also posted paper copies of the worksheets to their home address before the intervention start date, in case they preferred to use printed worksheets. Each participant also received weekly one-to-one telephone check-in calls with the practitioner psychologist (15 minutes duration) to support skills practice between sessions and provide an opportunity to reflect on the content covered in group sessions.

#### One-to-One Psychological Support

Participants considered to have moderate to high levels of emotional eating, or moderate to high levels of weight-related psychological distress, or symptoms of binge eating, but who did not wish to participate in a group intervention or presented with psychological issues that would benefit from an alternative psychological intervention, were allocated to this intervention. The therapeutic intervention that participants received depended on their presentation and on the practitioner psychologist’s bespoke formulation. Third wave CBTs (eg, Acceptance and Commitment Therapy, Dialectical Behavioral Therapy, and Compassion Focused Therapy) were among the commonly used evidence-based interventions [57,59-71]. This intervention included around 10 one-to-one video consultations (50 minutes, range 7‐13) via Zoom (Zoom Communications Inc) with a practitioner psychologist. Following each session, any relevant educational materials or worksheets to complete between sessions were emailed to the participant.

#### Physical Activity Support

All participants were offered the opportunity to discuss their physical activity support needs with a physical activity specialist. This involved a one-to-one telephone consultation (30 minutes) to discuss baseline physical activity levels and set specific, achievable, and realistic goals to increase physical activity. Examples of individually tailored goals include “To go out for a walk 3x per week, for 15 min,” “To walk 1000 steps, every day*,”* or “To introduce an at-home seated activity session 2‐3 times per week.” Following this consultation, participants were invited to attend weekly live group physical activity classes (30 minutes) via Zoom, facilitated by a physical activity associate (supervised by the physical activity specialist). A follow-up consultation to review progress was offered to all participants who completed phase 1 of the service.

### Health Care Professional Training

Health coaches delivering the psychologically informed app-based intervention included a registered nutritionist and an associate nutritionist, both registered with the UK Voluntary Register of Nutritionists (UKVRN), with experience of working with complex weight management patients in clinical settings. Health coaches were supervised by a dietitian registered with the Health and Care Professions Council (HCPC). Health coaches completed a comprehensive training intervention, which was delivered by a dietitian and practitioner psychologists with extensive experience in specialist weight management, to ensure high-quality care in line with professional guidelines and clinical protocols. Health coaches also attended weekly clinical supervision led by a dietitian and supported by a practitioner psychologist.

Practitioner psychologists were Health Psychologists registered with the HCPC, both with extensive experience of working in specialist weight management and a special interest in third wave CBTs. Each practitioner psychologist received weekly or monthly clinical supervision with a more senior practitioner psychologist, depending on experience.

The physical activity associate (level 2 qualification for gym instructors) was supervised by the physical activity specialist (level 3 personal trainer) during live physical activity sessions. Both professionals were registered with the Chartered Institute for the Management of Sport and Physical Activity (CIMSPA).

### Data Collection and Measures

#### Overview

Primary outcomes were weight change (kg) and percentage weight change at postintervention. Secondary outcomes included psychological distress, emotional eating, health-related quality of life, physical activity, intervention feasibility, and acceptability. For those who took part in the group-based intervention, an additional secondary outcome included emotion regulation.

Baseline characteristics (weight, height, age, gender, and type 2 diabetes diagnosis) and contact details (including postcode) were collected by the participant’s referrer and emailed securely via an NHS email service to Second Nature. Data were entered into Second Nature’s referral management system, and participants were sent an email link to complete a series of validated questionnaires about their current psychological distress, eating behaviors, health-related quality of life, and physical activity. Postcode data were also collected during onboarding to calculate socioeconomic deprivation based on the index of multiple deprivation (IMD). At the completion of the 16-week interventions, participants were sent another email link to complete postintervention questionnaires to measure intervention efficacy.

#### Effectiveness

##### Weight Change

Participants’ baseline weight (kg) and height (m) were recorded by the referring NHS SWMS on a referral form. Postintervention weight was self-reported via a questionnaire. No other weight data were collected during the intervention. Weight change data were calculated by subtracting postintervention weight from baseline weight, and participants were assigned to weight change categories of “lost,” “maintained,” and “gained.” Percentage weight loss was computed as: (weight lost or baseline weight) x100. Participant baseline and 16-week BMI was calculated with the formula BMI=kg/m^2^.

##### Psychological Distress

Th*e* Clinical Outcomes in Routine Evaluation–Outcome Measure (CORE-OM) [72] was used to evaluate psychological distress and consisted of 4 domains (subjective well-being, problems or symptoms, life functioning, and risk or harm) measured using 34 items. Each item was scored on a 0‐4 Likert scale, with higher scores indicating greater distress. Scores for each CORE-OM domain were calculated by summing the relevant item responses, yielding total scores that reflect cumulative distress within each area. Internal consistency of the CORE–OM has been reported as *α*=.94 [73].

##### Emotional Eating

The Emotional Eater Questionnaire (EEQ) [74] was used to assess emotional eating using 10 items, divided into 3 domains: disinhibition (loss of control over eating), types of food consumed in emotional situations, and guilt related to eating behaviors. Items are scored on a 4-point scale (0=Never, 3=Always), resulting in a total score ranging from 0‐30. Scores were interpreted as: nonemotional eater (0‐5), low emotional eater (6-10), emotional eater (11-20), very emotional eater (21-30). Internal consistency of the EEQ, within the context of obesity specifically, has been reported as *α*=.64 [74].

##### Health-Related Quality of Life

The EuroQol 5 Dimensions, 5 Levels (EQ-5D-5L) [75] was used to measure health-related quality of life through 5 dimensions: mobility, self-care, usual activities, pain and discomfort, and anxiety and depression. Each domain was rated out of 5 levels (1=no problems, 5=extreme problems). Responses generate a 5-digit health state code (eg, 11,111 for full health across the 5 domains). A self-rated measure of current perceived health on a scale of 0 (worst imaginable health) to 100 (best imaginable health) is also included in the form of a Visual Analog Scale (VAS). The internal consistency of the EQ-5D-5L has been reported as *α*=.79 [76].

##### Physical Activity

The General Practice Physical Activity Questionnaire (GPPAQ) [77] was used to measure and categorize physical activity into 4 levels (Active, Moderately Active, Moderately Inactive, and Inactive) based on 3 items: occupational activity, weekly physical activity, and walking pace. The range of internal consistency of the GPPAQ has been reported as *α*=.86‐0.97 [78]. Participants scoring below “Active” are recommended for a brief intervention to increase physical activity, in accordance with NICE guidelines [79].

##### Emotion Regulation

The Difficulties in Emotion Regulation Scale (DERS-16) [80] measures emotion regulation difficulties across 5 domains (nonacceptance of negative emotions, inability to engage in goal-directed behaviors when distressed, difficulties controlling impulsive behaviors when distressed, limited access to emotion regulation strategies perceived as effective, and lack of emotional clarity). Total scores range from 16‐80, with higher scores indicating greater difficulties in emotion regulation. This measure was completed only by participants allocated to the DBT-based skills training group intervention, as one of the aims of this intervention was to improve emotion regulation. The 16 items are scored on a 1‐5 scale (1=Almost never, 5=Almost always), producing a total score from 16‐80. Higher scores indicate greater difficulties with emotion regulation. The internal consistency of the DERS-16 has been reported as *α*=.92 [80].

### Feasibility

#### Retention and Discharge Reasons

Participants were discharged if they requested to withdraw or disengaged from the intervention (judged on a case-by-case basis) or no longer met the eligibility criteria (eg, pregnancy disclosure and cancer diagnosis). Reasons for discharge were recorded in a referral management system and leads of the referring SWMSs were informed of discharges.

#### Engagement

Across all 3 interventions, engagement was primarily measured through session attendance, including one-to-one and group sessions, and scheduled check-ins. In the app-based intervention, home screen views were used as a proxy for minimal digital engagement, reflecting user-initiated interaction with the app’s central hub. While this is a broad measure, it captures instances where participants accessed the platform to engage with key features such as modules, goals, or coach messages.

### Acceptability

A mixed-methods questionnaire was sent to all participants upon completion of their intervention to evaluate intervention acceptability and inform future service improvement. The questionnaire measured patient satisfaction ratings in relation to overall intervention experience on a 10-point Likert scale (1=Extremely dissatisfied, 10=Extremely satisfied), as well as providing free-text response options to qualitatively explore perceived benefits and challenges (What, if any, were the benefits and challenges of the program for you?), most and least useful program components (Which elements of the program did you find most and least useful?), self-reported behavior changes (What changes, if any, have you made as a result of the program?), and suggestions for service improvement (Was there anything you felt was missing from the overall program? and Based on your experience, how can we improve the program for future participants?).

### Statistical Analysis

Statistical analysis was carried out using IBM SPSS Statistics (V.29). Descriptive statistics were used to analyze participant demographics, which were reported in mean, SD, and range for continuous variables and in number and percentages for categorical variables. Descriptive statistics were used to assess feasibility measures. The attrition rate was calculated by dividing the number of noncompleters by the total number of referrals received, multiplied by 100, and missing data were managed through listwise deletion [81]. Normality of data distribution was assessed by performing Shapiro-Wilk tests (recommended for sample size <50 [82]) and Q-Q plots for each outcome variable, split by intervention allocation.

Within-group analyses on mean change from baseline to 16-week follow-up were used to analyze the potential effectiveness of interventions. Two-tailed paired-samples *t* tests were used for continuous variables and McNemar nonparametric tests were used for categorical variables. ANOVA tests were performed to evaluate between-group differences between interventions at baseline and at 16 weeks. Parametric and nonparametric correlational analysis was used to investigate relationships between outcome variables.

A priori power analysis for sample size estimation was carried out using G*Power (v.3.1; Heinrich Faul, Edgar Erdfelder, Axel Buchner, and Albert-Georg Lang) software [83] for both a paired-samples *t* test (for within-group weight change) and a one-way ANOVA (for between-group comparisons). With a significance criterion of *α*=.05 and 95% power for detecting a medium effect (d=0.5; *f*=0.25), the minimum sample size needed for a size was n=36. The calculation was based on the primary outcome of weight change at 16 weeks and was in line with Cohen benchmarks [84] and supported by prior meta-analyses of behavioral weight management interventions [85]. We acknowledge that smaller effects (d=0.3‐0.4) may be present in secondary outcomes, and that our sample size may limit power to detect these.

### Qualitative Analysis

Content analysis was conducted to analyze questions with free-text responses on the mixed-methods postintervention questionnaire. This approach involved categorizing free-text data into themes through a systematic process of coding and interpretation [86]. For each question, the responses were coded and organized into higher-level categories by GS. For example, perceived benefits were characterized by explicit benefits or positive outcomes related to the intervention, such as reduced binge eating episodes or decreased snacking frequency. Participants’ responses were organized into more than one category, where appropriate (eg, least useful intervention component and suggestion for service improvement). The codes and categories were then discussed with RR and refined. Finally, the frequency of occurrence of each category was calculated. Example quotes from participants are presented to illustrate key themes.

### Ethical Considerations

The Health Research Authority (HRA) decision tool was used to determine the nature of this study (Multimedia Appendix 1) [40]. This study met the criteria for service evaluations, as outlined by the HRA and Medical Research Council (1) it was designed and conducted solely to assess current care; (2) it evaluated a current service without reference to a standard; (3) it involved an intervention already in use; (4) it involved analysis of existing data; (5) there was no allocation to the intervention; and (6) there was no randomization, and therefore it did not require ethical approval (Multimedia Appendix 1) [40]. The data analyzed in this evaluation were not collected with the intent to conduct research, but to assess and improve service delivery. Therefore, this project does not constitute research under UK policy. The HRA explicitly states that "because both audit and service evaluation are considered part of usual professional practice, they are exempt from oversight processes that govern research, that is, they do not require review by a research ethics committee (REC)” [87]. As such, NHS REC/IRB review was not required for this study.

## Results

### Baseline Characteristics

A total of 39 participants were included in this evaluation (Table 1), of which 82.1% (n=32) were female, with a mean BMI of 46.8 kg/m^2^ (SD 9.04). Only 3 participants (7.7%) had a diagnosis of type 2 diabetes.

**Table 1.** Baseline characteristics of intervention participants.

Baseline sample characteristics, (N=39)	Values
Age (years), mean (SD, range)	45.7 (11.7, 26‐68)
Female sex, n (%)	32 (82.1)
Weight (kg), mean (SD)	132.8 (27.6)
BMI (kg/m^2^), mean (SD)	46.8 (9.0)
Presence of type 2 diabetes, n (%)	3 (7.7)
IMD^a^, n (%)	
1 (most deprived)	0 (0)
2	7 (17.9)
3	16 (41.0)
4	13 (33.3)
5 (least deprived)	2 (5.1)

a IMD: index of multiple deprivation

### Intervention Allocation

Following the initial assessment process, participants were allocated to one of 3 interventions. Almost half (19/39, 48.7%) of participants were allocated to the app-based intervention, while participants allocated to the psychological interventions were equally split between group-based (10/39, 25.6%) and one-to-one (10/39, 25.6%).

### Effectiveness

#### Weight Change

There was a statistically significant difference in total sample weight change at 16 weeks (*t*_38_=2.64; *P*=.01) with a medium effect size (Cohen *d*=0.42), but no significant change in BMI (*t*_38_=0.85; *P*=.40). Average weight change postintervention was −2.2kg (−1.6%, SD 5.16). Mean baseline and change scores at 16 weeks for weight and BMI are reported in Table 2, including differences between interventions.

Participants in the app-based intervention lost the most weight at postintervention (−2.8kg), and participants receiving one-to-one psychological support lost the least weight (−1.3kg), although no significant between-group differences in weight and BMI were observed at baseline (*F*_2,36_=.64; *P*=.53 and *F*_2,36_=.39, *P*=.68 respectively) or at postintervention (*F*_2,36_=.81; *P*=.45 and *F*_2,36_=.51; *P*=.61, respectively).

A total of 46.2% (n=18) of participants lost weight, 35.9% (n=14) maintained baseline weight, and 17.9% (n=7) gained weight at postintervention. No significant correlations were observed between weight changes at post-intervention and changes in any of the other secondary outcome variables, with the exception of the EEQ, where weight loss was significantly positively correlated with a reduction in emotional eating behavior (*r*_39_=.347, *P*=.03).

**Table 2.** Average weight and BMI change at 16 weeks.

Intervention	Baseline		16-week	
	Weight (kg), mean (SD)	BMI (kg/m^2^), mean (SD)	Weight change (kg), mean (%, SD)	BMI change (kg/m^2^), mean (SD)
Psychologically informed, app-based intervention	128.0 (25.0)	45.5 (6.93)	−2.8 (-2.2, 4.74)	−0.9 (3.27)
DBT-based^a^ skills training group intervention	134.4 (33.2)	48.3 (12.0)	−1.9 (-1.4, 6.30)	−0.7 (2.36)
One-to-one psychological support	140.1 (27.5)	47.9 (9.85)	−1.3 (-0.9, 5.11)	−0.5 (2.51)
Total (N=39)	132.7 (27.6)	46.8 (9.04)	−2.2 (-1.7, 5.16)^b^	−0.39 (2.89)

a DBT: Dialectical Behavioral Therapy.

b *P*<.05

#### Psychological Distress

At baseline, the total sample mean score was 1.4 (SD 0.68), where 71.8% (n=28) of participants scored above the threshold (mean score=1) for clinically significant psychological distress. The correlation between psychological distress and baseline weight approached statistical significance (*r*_39_=.311; *P*=.05). At 16 weeks, 46.2% (n=18) of participants scored above the threshold and the total sample mean score was 0.95 (SD 0.62). This reduction (−25.6%) was statistically significant (*t*_38_=5.35; *P*<.001), with a large effect size (Cohen *d*=0.86) (). There were also significant improvements in individual CORE-OM domains of subjective well-being (*t*_38_=5.09; *P*<.001), problems and symptoms (*t*_38_=4.94; *P*<.001), and life functioning (*t*_38_=5.18; *P*<.001). Mean risk or harm domain scores decreased by −0.36 (SD 1.55) at 16 weeks; however, this was not statistically significant (*t*_38_=1.45, *P*=16).

Participants allocated to the DBT-based group intervention and one-to-one psychological support showed higher baseline CORE-OM mean scores (mean 1.66, SD 0.59 and mean 1.59, SD 0.74, respectively) than those allocated to the app-based intervention (mean 1.14, SD 0.64); however, these differences were not statistically significant (*P*=.08). Similarly, there were no significant between-group differences in CORE-OM mean scores at 16 weeks (*P*=.35) , nor in the domains of subjective well-being (*P*=.40), problems and symptoms (*P*=.17), life functioning (*P*=.52) or risk or harm (*P*=.65) (Multimedia Appendix 2).

#### Emotional Eating

At baseline, 38.5% (n=15) of participants were categorized as “Very emotional eaters”, 41% (n=16) as “Emotional eaters,” 17.9% (n=7) as “Low emotional eaters”, and 2.6% (n=1) as “Nonemotional eaters”. At 16 weeks, a reduction in emotional eating severity was observed, with fewer participants categorized as “Very emotional eaters” (6/39, 15.4%) and “Emotional eaters” (14/39, 35.9%), and more participants categorized as “Low emotional eater” (15/39, 38.5%) and “Nonemotional eaters” (4/39, 10.3%).

The mean EEQ score at baseline was 17.6 (SD 7.20), which reduced to 12.3 (SD 6.81) at 16 weeks (Emotional eater category range 11‐20). This reduction in emotional eating behavior was statistically significant (*t*_38_=5.21; *P*<.001), with a large effect size (Cohen *d*=0.83).

Table 3 shows the breakdown of EEQ score changes by intervention. Participants in the DBT-based group intervention displayed the highest levels of emotional eating, and participants in the app-based intervention displayed the lowest. These between-group differences were statistically significant at both baseline (*F*_2,36_=10.42; *P*<.001) and 16-week (*F*_2,36_=8.48; *P*<.001). The greatest improvement in emotional eating behaviors was seen in the app-based intervention (*t*_18_=3.89; *P*=.001) at 16 weeks, while there was no significant change observed for those allocated to one-to-one psychological support (*P*=.13).

**Table 3.** Average emotional eating scores at baseline and 16-week, with EEQ score categorization.

Intervention	Baseline, mean (SD)	EEQ^a^ score category	16-week, mean (SD)	EEQ score category
Psychologically informed, app-based intervention	13.74 (6.06)	Emotional eater	8.53 (4.25)^b^	Low emotional eater
DBT-based^c^ skills training group intervention	24.2 (5.43)	Very emotional eater	17.3 (7.35)^d^	Emotional eater
One-to-one psychological support	18.2 (5.98)	Emotional eater	14.4 (6.57)	Emotional eater
Total (N=39)	17.6 (7.20)	Emotional eater	12.3 (6.81)^b^	Emotional eater

a EEQ: Emotional Eating Questionnaire

b *P*<.001

c DBT: Dialectical Behavioral Therapy.

d *P*<.05

#### Health-Related Quality of Life

At both baseline and 16-week, participants had on average, slight-to-moderate perceived problems with their mobility, usual activities, pain and discomfort, and anxiety and depression, and no-to-slight problems with self-care. While postintervention scores decreased, particularly for self-care, usual activities, and anxiety and depression (respectively: *t*_38_=2.08, *P*=.04; *t*_38_=2.58, *P*=.01; *t*_38_=2.27, *P*=.03), the reductions were not sufficient to move participants into categories of lower perceived severity of problems. Participants’ challenges with mobility and pain and discomfort remained unaffected (*P*>.05). Table 4 shows domain-specific mean scores and frequencies, alongside *t* test *P* values.

**Table 4.** EuroQol 5 Dimensions, 5 Levels domain mean scores, response distribution by severity level, and *P* values from comparison of means analysis.

EQ-5D-5L^a^ domain	Score, baseline		16-week score		*P* value^b^
	Score, mean (SD)	Participants (N=39), n (%)	Score, mean (SD)	Participants (N=39), n (%)	
Mobility	2.64 (1.25)	—^c^	2.41 (1.23)	—	.12
No problems	—	10 (25.62)	—	13 (33.32)	—
Slight problems	—	8 (20.50)	—	8 (20.50)	—
Moderate problems	—	8 (20.50)	—	7 (17.93)	—
Severe problems	—	12 (30.81)	—	11 (28.22)	—
Unable to walk about	—	1 (2.63)	—	0 (0)	—
Self-care	1.85 (1.18)	—	1.54 (0.88)	—	.04^d^
No problems	—	22 (56.4)	—	27 (69.22)	—
Slight problems	—	8 (20.50)	—	4 (10.31)	—
Moderate problems	—	3 (7.71)	—	7 (17.93)	—
Severe problems	—	5 (12.81)	—	1 (2.63)	—
Unable to wash or dress	—	1 (2.63)	—	0 (0)	—
Usual activities	2.44 (1.17)	—	2.03 (0.96)	—	.01^d^
No problems	—	9 (23.1)	—	14 (35.92)	—
Slight problems	—	14 (35.9)	—	13 (33.32)	—
Moderate problems	—	8 (20.50)	—	9 (23.12)	—
Severe problems	—	6 (15.44)	—	3 (7.73)	—
Unable to do usual activities	—	2 (5.11)	—	0 (0)	—
Pain and discomfort	2.87 (1.20)	—	2.82 (1.10)	—	.70
No problems	—	5 (12.81)	—	3 (7.73)	—
Slight problems	—	12 (30.81)	—	15 (38.51)	—
Moderate problems	—	8 (20.50)	—	10 (25.62)	—
Severe problems	—	11 (28.22)	—	8 (20.50)	—
Extreme pain or discomfort	—	3 (7.73)	—	3 (7.73)	—
Anxiety and depression	2.59 (1.12)	—	2.23 (1.09)	—	.03^d^
No problems	—	9 (23.12)	—	12 (30.81)	—
Slight problems	—	7 (17.93)	—	11 (28.22)	—
Moderate problems	—	15 (38.51)	—	13 (33.32)	—
Severe problems	—	7 (17.93)	—	1 (2.63)	—
Extremely anxious or depressed	—	1 (2.63)	—	2 (5.11)	—

a EQ-5D-5L: EuroQol 5 Dimensions, 5 Levels

b Within group differences.

c Not applicable.

d *P*<.05

There were no between-group differences at baseline for participants’ scores in pain and discomfort, or anxiety and depression (*P*>.05); however, participants allocated to the app-based intervention showed significantly lower scores for mobility (*F*_2,36_=3.43, *P*=.04), self-care (*F*_2,36_=3.51, *P*=.04) and usual activities (*F*_2,36_=3.83, *P*=.03), indicating fewer perceived problems in each domain. Participants allocated to one-to-one psychological support showed the highest levels of perceived problems for mobility (mean 3.30, SD 1.2) and usual activities (mean 3.0, SD 1.1).

Subjective ratings of health status also improved at 16 weeks (*t*_37_=−3.18; *P*=.003), with a medium effect size (Cohen *d*=0.52), with participants rating on average 53.5/100 at baseline and 62.8/100 at 16 weeks, indicating a 17.4% improvement in subjective health status. There were no between-group differences in subjective health status at baseline (*F*_2,35_=2.57, *P*=.09) or 16-week (*F*_2,36_=2.30, *P*=.12); however, participants allocated to the app-based intervention subjectively rated their health higher (mean 61.3, SD 22.2) than those in the DBT-based group intervention and one-to-one psychological support (mean 44.9, SD 19.3, and mean 48, SD 19.9, respectively) at baseline (Table 5). Despite participants in the DBT-based group intervention experiencing the greatest improvements at 16 weeks (26.7% increase), only the app-based intervention resulted in significant postintervention change (*t*_17_=−2.93, *P*=.009).

**Table 5.** Average subjective ratings of health status at baseline and 16-week (out of 100).

Intervention	Baseline, mean (SD)	16-week, mean (SD)	*P* value
Psychologically informed, app-based intervention	61.3 (22.21)	70.0 (18.64)	.009^a^
DBT-based skills training group intervention	44.9 (19.32)	56.9 (19.92)	.14
One-to-one psychological support	48.0 (19.91)	56.3 (20.81)	.28
Total (N=39)	53.5 (21.33)	62.8 (20.32)^a^	.00^a^

a *P*<.05

#### Physical Activity

At baseline, 61.5% (n=24) of participants were categorized as “Inactive”, 17.9% (n=7) as “Moderately inactive,” 15.4% (n=6) as “Moderately active,” and 5.1% (n=2) as “Active.” No significant changes in physical activity levels were observed at 16-week (*P*=.26). The majority of participants (35/39, 84.6%) attended a call with the physical activity specialist at baseline and were supported to set individualized physical activity goals, while only 4 participants attended the live activity classes, with an average of 5 sessions per participant (range 1‐16). Only 2 participants opted in for a follow-up call with the physical activity specialist to review their goals at 16 weeks.

#### Emotion Regulation

Participants allocated to the DBT-based group intervention scored a mean DERS-16 score of 50.8 (SD 16.8) at baseline and a mean score of 39.2 (SD 13.2) at 16-week, with a mean change score of −11.6 (SD 13.9). This reduction in difficulties in emotion regulation was statistically significant (*t*_9_=2.63; *P*=.03).

### Feasibility

#### Retention and Discharge Reasons

Out of a total of 62 referrals received, 10 participants were discharged before starting the intervention for the following reasons: need to prioritize other physical or mental health support needs over weight management goals (n=2), low digital literacy (n=1), missed video consultation appointments (n=2), did not complete baseline assessment questionnaires (n=1), and other (n=4). A further 7 participants were discharged during the intervention due to low or no engagement or changes in life circumstances which impacted their ability to engage with the intervention. Outcomes from 6 further participants were not yet available at the time of analysis. The attrition rate was 27.4% (n=7).

#### Engagement

On average, participants attended 93.7% of the intervention sessions, with the highest attendance for the app-based intervention (96.1%) and the lowest for the DBT-based group intervention (87.5%). Session attendance was a mean 7.7 (out of 8) sessions (SD 0.58, range 6‐8) for the app-based intervention, 14 (out of 16) sessions (SD 1.56, range 11‐16) for the DBT-based intervention, and 11 sessions (SD 1.56, range 7‐13) for one-to-one psychological support. Participants in the app-based intervention attended on average 4.1 (out of 8) in-app check-ins (SD 2.90, range 0‐8) and participants in the DBT-based group intervention attended on average 11.7 (out of 16) telephone check-in calls (SD 2.82, range 8‐16).

No significant correlation between session attendance (%) and any of the primary and secondary outcome variables was observed (*P*>.05).

Participants in the app-based intervention consistently engaged with the app throughout the 16 weeks; however, home screen views showed a slight decrease from baseline (mean 4.52, SD 1.13) to Week 16 (mean 3.03, SD 1.29)

### Acceptability

The postintervention acceptability questionnaire was completed by 56.4% (n=22) of participants, all of whom provided responses to each of the free-text questions. Overall, participants’ experiences of the interventions were rated at an average 9 out of 10, with one-to-one psychological support showing higher satisfaction ratings (mean 9.8/10, SD 0.92) than the app-based intervention and the DBT-based group intervention (mean 8.8/10, SD 0.53 and mean 9/10, SD 1.20, respectively).

*Having a working relationship that felt I was respected and an equal member in my treatment.*

[Participant 1, one-to-one psychological support intervention]

Overall, the main reported benefits of the interventions were behavioral changes (14/22, 63.6%) (eg, reduced binge eating episodes, decreased snacking frequency), psychological improvements (12/22, 54.5%) (eg, development of emotion regulation strategies and other coping mechanisms), educational value (11/22, 50%) (eg, improved understanding of weight loss barriers and personal eating habits), strong support system (9/22, 40.9%) (eg, meaningful relationships with peers and intervention facilitators and normalization of common challenges), and practical skills development (8/22, 36.4%) (eg, goal setting and improvements in managing cravings and urges).

*I feel much more empowered to cope/manage emotions and feel hopeful for reaching my goals*.

[Participant 2, DBT-based group intervention]

With regard to challenges of the interventions, participants reported struggling with psychological barriers ( 9/22, 40.9%) (eg, connection between eating-related distress and recollection of experiences of trauma or adversity), as well as time-related difficulties (8/22, 36.4%) (eg, frequency or intensity of intervention commitments, and complexity of assessment processes).

*To accept that trauma has shaped my reactions to everyday occurrences, and to identify my emotions.*

[Participant 3, one-to-one psychological support intervention]

The main self-reported behavior changes included dietary changes (13/22, 59.1%) (eg, implementation of the “balanced plate approach” [88] and reduction in portion sizes and snacking behavior); implementation of psychological strategies (10/22, 45.5%) (eg, challenging all-or-nothing thinking and self-criticism, more effective management of triggers to emotional distress); and interpersonal communication strategies (5/22, 22.7%) (eg, more effective and assertive strategies to communicate eating-related challenges with family and friends).

*Eating more fruit and vegetables! Decision fatigue was a major barrier for me, I now have a few options for each meal and choose throughout the week. I accept that I can have a bit of chocolate but I don’t have to eat as much, I'm more mindful! I've become more active, I enjoy the gym now!*

[Participant 4, app-based intervention]

The most useful elements of the intervention reported by participants were one-to-one support (14/22, 63.6%), educational components (10/22, 45.5%), peer support (6/22, 27.3%), and overall intervention structure (5/22, 22.7%), while the elements considered least useful were difficulty with information retention in text-heavy educational material (5/22, 22.7%), app-related technical issues (3/22, 13.6%), and the physical activity live classes at unsuitable times (7/22, 31.8%).

*Been able to have conversations with people regarding trigger factors and learnt to sit with the thoughts and be okay with not being okay.*

[Participant 5, app-based intervention]

The main suggestions from participants for future improvements included app functionality (5/22, 22.7%) (eg, better notification system), content delivery methods (4/22, 18.2%) (eg, reduced repetition in course structure and improved recipe option), and accessibility (3/22, 13.6%) (eg, increased appointment scheduling flexibility and reducing text-heavy format of some of educational material).

*More of an issue with the app but I didn't ever receive notifications which I would have liked to remind me to check in etc.*

[Participant 6, app-based intervention]

## Discussion

### Overview

This study aimed to evaluate the potential effectiveness, feasibility, and acceptability of a remotely delivered SWMS for patients living with obesity, referred from NHS SWMSs in the United Kingdom. The study showed promising outcomes in weight change, psychological distress, emotional eating behavior, perceived health-related quality of life, and emotion regulation following the first phase (16 weeks) of a 3-phase service. While challenges remain, such as low engagement in physical activity, the present findings provide a strong foundation for the role of remotely delivered SWMSs in addressing current gaps in obesity service provision in the United Kingdom.

### Principal Findings

The majority of participants had either lost or maintained weight upon completion of their intervention. This is a positive finding given that the aim of this first phase of the service (Preparing for weight loss) was to first address psychological barriers to behavior change to support weight loss in subsequent phases, rather than prioritizing immediate weight reduction. In line with this innovative approach, the NIH emphasizes the prevention of further weight gain as a treatment target in order to mitigate associated health risks with obesity, which manifests as a chronic and relapsing condition [89,90]. More recently, a review of the definition and diagnostic criteria of clinical obesity suggested prioritizing obesity symptom management as an outcome (such as improvements in metabolic health, psychological distress, and quality of life) rather than weight loss, highlighting the importance of integrating psychological support into weight management interventions to enhance outcomes for people living with obesity [10,60].

Notably, despite the complexity of the present cohort, characterized by high body weight, high levels of psychological distress, and significant challenges with mobility and daily activities, participants still achieved positive weight outcomes. This suggests that a health-centric (eg, prioritizing the addressing of psychological barriers), rather than a weight-centric (eg, prioritizing weight loss) approach can be an effective strategy to support weight loss in this population group and that psychologically informed DWMIs have the potential to set the stage for long-term sustainable weight management [10,41,59,68].

Unsurprisingly, the psychologically informed app-based intervention saw greater weight change at 16 weeks compared to the DBT-based group intervention and one-to-one psychological support. This is likely explained by the higher degree of psychological complexity and barriers to change of participants allocated to the DBT-based group intervention and one-to-one psychological support, as supported by existing literature. Studies indicate that individuals with elevated psychological distress, comorbid mental health conditions, or difficulties in emotion regulation often face greater challenges in initiating and sustaining behavioral changes due to reduced motivation, increased difficulties with emotional regulation, and lower self-efficacy [91,92]

The majority of participants began the intervention with clinically significant levels of psychological distress, which is a common presentation in populations living with obesity and multiple comorbidities [93-95]. The finding that baseline psychological distress was higher in those allocated to the DBT-based group intervention and one-to-one psychological support suggests the appropriateness and feasibility of the assessment process used to allocate participants to their interventions. Equitable distribution of improvements in psychological distress across interventions further supports the effectiveness of tailoring interventions to individual needs.

At 16 weeks, psychological distress scores decreased below the clinical threshold and improvements were observed across 3 scale domains, suggesting comprehensive psychological benefits and alleviation of psychological symptoms associated with obesity. It is important to note that this clinically significant improvement occurred without a significant reduction in body weight across groups.

While improvements in psychological distress did not directly correlate with weight change, participants who reduced emotional eating behaviors demonstrated greater weight loss. The interventions successfully reduced emotional eating behaviors, highlighting the potential efficacy of the psychological intervention components in addressing maladaptive eating patterns. This is supported by research demonstrating that emotional eating is a key contributor to obesity and that interventions focusing on emotion regulation and psychological support can lead to significant reductions in both emotional eating and overall weight [96,97]. Furthermore, studies have suggested that emotional eating behaviors could mediate the relationship between psychological distress and weight maintenance in a population with a history of weight cycling [91,98,99], and that long-term weight loss maintenance outcomes are closely linked to improvements in emotional eating behaviors [96,100].

Participants allocated to the DBT-based group intervention reported moderate baseline difficulties in emotion regulation, which decreased at 16 weeks, indicating progress in managing emotions and possibly contributing to a reduction in emotional eating behaviors. These findings are noteworthy given the relationship between psychological distress and psychological barriers to weight management, such as distress management, emotional eating, and reduced self-efficacy [101,102].

There were improvements in perceived health-related quality of life with regards to increased self-care practices, better ability to perform usual activities, and reduced anxiety and depression symptoms, suggesting that the interventions positively influenced functional independence and emotional well-being. Behavioral weight management interventions have previously been shown to lead to enhancements in a range of health-related outcomes, including reductions in depressive symptoms and improvements in quality of life [103].

However, perceived problems with mobility, and pain and discomfort remained unaffected. Generally, research indicates that while weight loss interventions may provide small to moderate improvements in pain and disability for conditions like osteoarthritis, these effects can be limited, suggesting the need for more tailored and prolonged interventions to effectively address these domains [104].

Nonetheless, the observed improvement in subjective health ratings reflects participants’ perception of enhanced overall health, despite their complex medical and psychological presentations, and is comparable to those of other studies [23]. Participants who accessed the DBT-based group intervention experienced the largest improvements in perceived subjective health rating, possibly due to DBT’s focus on balancing acceptance with change as a meaningful therapeutic approach to promote changes in both emotional and behavioral domains, as well as improvements in perceived health, as further evidenced by other studies [105,106]. It is also possible that the group nature of the DBT-based intervention contributed to improvements in subjective health ratings at 16 weeks, given that social support has been identified as a key component for weight management interventions effectiveness [107-109], even in the context of remotely delivered interventions [110,111].

The finding that no significant changes in physical activity levels were observed reflects the challenges of increasing physical activity in people living with obesity who typically experience significant physical and mental health comorbidities [112,113]. Despite high baseline engagement with physical activity-related goal setting, engagement with follow-up calls and live activity class participation was limited, and overall physical activity levels did not increase at post-intervention. A recent systematic review identified competing priorities and time constraints as key barriers to lifestyle change intervention adherence [114]; therefore, it is possible that participants engaged in the physical activity support less than anticipated due to a sense of being overwhelmed by the demands of their core intervention, with establishing new physical activity habits feeling more like an additional burden on top of their other commitments, rather than an opportunity to implement further behavior change. Another potential barrier could have been the limited flexibility in the days and times the live activity classes took place, restricted by the availability of the physical activity specialist and associate. This aligns with previous studies that highlight the need to mitigate the effects of time-related barriers to physical activity engagement in people living with obesity by providing flexible and time-efficient support offers [112]. The remotely delivered nature of the live activity classes could have also acted as an engagement barrier, in line with findings from other studies which suggested that adherence to home-based physical activity interventions is often low in unsupervised settings with limited-to-no face-to-face activity supervision [115-117].

Retention in lifestyle change interventions is often challenging, with attrition rates in in-person weight management interventions averaging around 50% [118,119]. Nonetheless, intervention tailoring to individual needs, convenience of setting, and more acceptable service design can greatly improve participant retention, as suggested by an evaluation of a SWMS based in primary care [23]. Attrition rates of this study fall within the range of average attrition rates in DWMIs at around 12 weeks (16%‐50%) [120,121]. Factors influencing completion of DWMIs include lack of human interaction, lower baseline motivation, lower levels of digital literacy, and technical challenges. High attendance rates throughout the present study’s interventions potentially reflect effective intervention design and intervention allocation, face-to-face support via video calls, and tailoring, which align with factors identified as reducing dropout rates and improving service feasibility [121,122]. Consistent patterns in app home screen views throughout the 16-week further support these findings, suggesting sustained digital engagement that likely contributed to the observed attrition and attendance rates across interventions.

Participants reported very high levels of satisfaction with their interventions, averaging 9 out of 10, with one-to-one psychological support receiving the highest ratings. Furthermore, participants highlighted key intervention benefits, including behavioral changes, psychological improvements, and educational value. Strong support from facilitators and peers was also valued by participants. Challenges included psychological barriers, such as trauma-linked distress, text-heavy resources, and time demands of the intervention. Suggestions for improvement focused on the app notification system, more engaging content delivery, and increased scheduling flexibility. These findings align with other studies that saw similar satisfaction ratings and overall acceptability outcomes within the context of remotely delivered weight management interventions [123]. For example, a technology-based, rural weight management intervention in older adults with obesity identified behavioral changes as the main perceived benefits, and other existing health conditions (including mental health conditions) and technological challenges as the main perceived barriers [122].

### Limitations

This study was a service evaluation that used retrospective data in the absence of a control group and randomization. Therefore, it is not possible to draw definitive conclusions about the effectiveness of the intervention nor compare intervention efficacy to standard care outcomes.

Collection of reliable weight data in SWMSs is a common challenge [124,125]. Some participants reported their baseline weight data (received upon referral) to be out of date and, at times, lower than their current weight. Weight data at 16 weeks was self-reported [126], with some participants only able to provide weight estimates, particularly when they opted not to weigh themselves to avoid psychological distress and associated potential triggers of emotional eating, or when self-weighting equipment was unavailable or inaccessible as can often be the case for people living with high body weights or mobility limitations [127,128]. It is possible the present study might have overestimated weight change outcomes due to a tendency of individuals, particularly women, to underreport weight data, often due to biases such as social pressure and self-esteem issues [124]. Improvement in the validity of weight data is needed to increase confidence in findings and provide a more accurate understanding of the impact of this pilot service on weight change.

It is important to note that the smaller sample sizes in the DBT-based group intervention and one-to-one psychological support limit the generalizability of the present findings; therefore, further analysis with larger sample sizes at completion of this 3-phase remotely delivered SWMS is needed. Furthermore, although the final total sample size exceeded the minimum requirements calculated via a priori power analysis, a larger sample would increase power to detect smaller effects in secondary outcomes, such as psychological distress and emotional eating—outcomes that may still be clinically meaningful despite modest effect sizes—and would better support further intervention improvement and tailoring.

In addition, while app home screen views were used as a proxy for digital engagement, this metric does not capture the extent to which participants actively engaged with specific intervention components, such as the educational modules. However, key module content was reinforced and discussed during one-to-one health coaching video calls, partially mitigating this limitation.

The modest response rate to the postintervention acceptability questionnaire may limit the representativeness of qualitative feedback, highlighting the value of collecting feedback earlier in the program to reduce participant burden and improve response rates.

Most participants were female, and no ethnicity data were collected, limiting conclusions drawn from the present analysis and its generalizability. Furthermore, despite the majority of participants residing in areas of low-to-moderate deprivation, most of them lived in rural and island communities, where the IMD is believed to underestimate the prevalence of deprivation and associated health inequalities [129].

### Conclusions

This preliminary service evaluation suggested that a remotely delivered SWMS has the potential to be effective, feasible, and acceptable for NHS-referred patients in the United Kingdom. The intervention showed promising improvements in psychological distress, emotional eating, perceived quality of life, and difficulties in emotion regulation and was effective in supporting weight stabilization and modest weight loss during the initial 16 weeks, particularly through psychologically informed components. Areas for further improvement were highlighted, including accuracy of primary outcome data collection, improvements in physical activity promotion, and a more representative sample diversity.

A planned comprehensive evaluation at 12 months will measure the same primary and secondary outcomes—weight change, psychological distress, emotional eating, perceived quality of life, and difficulties in emotion regulation—with a larger sample size to provide more robust, reliable, and generalizable findings. Qualitative feedback will also continue to be collected, to better understand participant experiences and intervention acceptability. More rigorous conclusions regarding the effectiveness, feasibility, and acceptability of remotely delivered SWMS interventions will be possible following this extended evaluation. These findings will also inform the scalability, future implementation, and directions for future research in this field.

## Supplementary material

10.2196/71914 Multimedia Appendix 1 Health research authority decision tool result.

10.2196/71914 Multimedia Appendix 2 Average psychological distress by intervention.

10.2196/71914 Checklist 1 SQUIRE (version 2.0) checklist.

## Abbreviations

CBT: cognitive behavioral therapy
CIMSPA: Chartered Institute for the Management of Sport and Physical Activity
CORE-OM: Clinical Outcomes in Routine Evaluation – Outcome Measure
DBT: Dialectical Behavioral Therapy
DERS-16: Difficulties in Emotion Regulation Scale
DWMI: Digital Weight Management Intervention
EEQ: Emotional Eater Questionnaire
EQ-5D-5L: EuroQol 5 Dimensions, 5 Levels
GPPAQ: General Practice Physical Activity Questionnaire
HCPC: Health and Care Professions Council
HRA: Health Research Authority
IMD: index of multiple deprivation
MDT: Multidisciplinary Team
NHS: National Health Service
NICE: National Institute for Health and Care Excellence
SQUIRE: Standards for Quality Improvement Reporting Excellence
SWMS: Specialist Weight Management Service
UKVRN: UK Voluntary Register of Nutritionists
VAS: Visual Analog Scale
