# Supplementary figures and images for: A Remotely Delivered Weight Management Service to Support Existing Obesity Services in the UK National Health Service: Preliminary Findings From an Early-Stage Service Evaluation
Source: JMIR Form Res. 2025 Oct 15;9:e71914. doi: 10.2196/71914 (PMC12527323; doi:10.2196/71914)

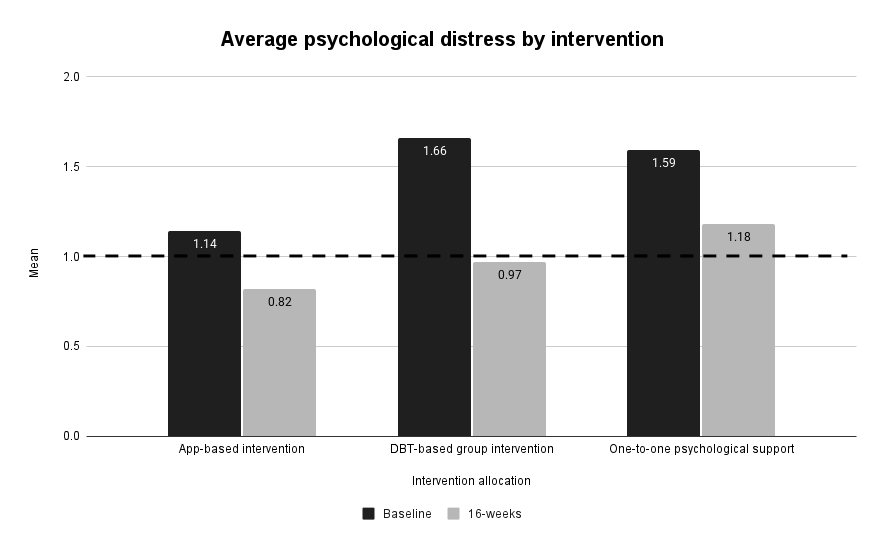

Supplement: Multimedia Appendix 2 [file formative-v9-e71914-s002.png]
