# Supplementary material for: A Remotely Delivered Weight Management Service to Support Existing Obesity Services in the UK National Health Service: Preliminary Findings From an Early-Stage Service Evaluation
Source: JMIR Form Res. 2025 Oct 15;9:e71914. doi: 10.2196/71914 (PMC12527323; doi:10.2196/71914)
Supplement: Checklist 1 [file formative-v9-e71914-s003.pdf]

SQUIRE 2.0 Checklist – Completed for Submitted Manuscript

Title: A remotely-delivered weight management service to support existing obesity services in the UK National Health Service: Preliminary findings from a service evaluation

Authors: Giulia Spaltro, Michael Whitman & Rebecca Richards

Section	Item	Checklist Description	Paper Location / Notes
Title and Abstract	1. Title	Indicates the manuscript concerns an initiative to improve healthcare	✓ The title clearly reflects the nature of the work as preliminary findings from a service evaluation of a healthcare improvement initiative delivered remotely within the NHS context.
Title and Abstract	2. Abstract	Provides adequate summary: background, local problem, methods, results, conclusions	✓ A structured abstract is included with headings for Background, Objective, Methods, Results, and Conclusions. It summarizes the study's context, aims, intervention, key outcomes, and implications.
Introduction	3. Problem Description	Nature and significance of the local problem	✓ The paper describes long NHS waiting times and limited access to specialist weight management services (SWMS) as a significant barrier to access to specialist care for patients living with obesity in the UK.
Introduction	4. Available Knowledge	Summary of current knowledge	✓ The introduction reviews existing literature on digital weight management

			interventions (DWMIs), including evidence from systematic reviews and recent UK-based studies, highlighting a gap in real-world NHS-integrated digital services.
Introduction	5. Rationale	Informal/explicit theory supporting intervention	✓ The intervention is grounded in behavior change theory (COM-B model) and psychological frameworks (e.g., DBT). The rationale is to address psychological barriers early in treatment to support sustainable weight-related behavior change.
Introduction	6. Specific Aims	Purpose of the project	✓ The aim is to evaluate the preliminary effectiveness, feasibility, and acceptability of the first phase of a three-phase remotely delivered SWMS for NHS-referred patients.
Methods	7. Context	Relevant elements of setting	✓ The service was implemented in partnership with existing NHS SWMS leads, with participants referred from NHS services across various UK regions, including rural areas.
Methods	8. Interventions	Detailed description of interventions	✓ Participants were allocated to one of three 16-week remote interventions based on a structured assessment: (1) app-based coaching, (2) dialectical-behaviour-therapy-based group skills training, or (3) one-to-one psychological support. Interventions

			were tailored and delivered by trained professionals.
Methods	9. Study of Interventions	Approach to assessing impact	✓The study used a retrospective service evaluation design with quantitative and qualitative outcome measures. Intervention fidelity was supported through structured training, supervision, and multidisciplinary team reviews. Engagement data were tracked to assess delivery consistency.
Methods	10. Measures	Chosen measures and rationale	✓ Includes validated measures for psychological and physical outcomes<br>✓ Quantitative outcomes (e.g., weight change, psychological distress, emotional eating) were assessed using validated instruments including CORE-OM, EEQ, EQ-5D-5L, GPPAQ, and DERS-16. Internal consistency (Cronbach's alpha) values were reported for each measure to demonstrate reliability.<br>✓ Qualitative data were collected from open-ended questions in the post-intervention acceptability survey to assess perceived benefits, challenges, and areas for improvement.
Methods	11. Analysis	Qualitative and quantitative methods	✓ Quantitative analysis included descriptive statistics, paired-samples t-tests, ANOVAs, and correlational analysis, using SPSS v29. Qualitative data were

			analyzed using content analysis: responses were coded, categorized into themes, and reviewed collaboratively by two authors to ensure consistency and rigor.
Methods	12. Ethical Considerations	Ethics, consent, and oversight	✓ This study is classified as a service evaluation according to the UK Health Research Authority (HRA) and is therefore exempt from NHS Research Ethics Committee (REC) review. The HRA explicitly states that service evaluations are part of usual professional practice and do not require REC oversight. This classification was confirmed using the official HRA decision tool, which serves as the recognized documentation of exemption. No identifiable data were used, and all data were anonymized and securely stored. A copy of the HRA decision tool result is included as **Multimedia Appendix 1**, in line with JMIR ethical guidelines.
Results	13. Results	Process and outcome data	✓ Results are presented clearly with appropriate statistics (e.g., means, SDs, p-values, effect sizes) for primary and secondary outcomes. Descriptive data, engagement metrics, attrition, and adherence are reported for each intervention arm.

			Qualitative findings from post-intervention survey responses are thematically analyzed and integrated to illustrate perceived benefits and challenges.
Results	14. Contextual Elements	Unexpected consequences or issues	✓ Participant complexity (e.g., comorbidities, psychological distress, trauma), digital literacy, and scheduling barriers influenced engagement. Broader service-level factors—such as NHS waiting list pressures and variability in SWMS capacity—also shaped referral flow and feasibility. Rural and remote settings introduced access challenges relevant to digital service delivery.
Results	15. Missing Data	Description and handling	✓ Missing data primarily related to self-reported post-intervention weight data and incomplete survey responses. Some baseline weight data may have been outdated or estimated. Data were handled using listwise deletion, and the potential impact on weight outcome validity is acknowledged in the manuscript.
Discussion	16. Summary	Key findings and relevance	✓ The study found that a remotely delivered SWMS was feasible, acceptable, and associated with modest weight loss and improvements in psychological outcomes. These findings align with the service's initial aim to assess early-phase

			effectiveness and address barriers to weight-related behavior change.
Discussion	17. Interpretation	Comparison with other studies	✓ Findings are interpreted in relation to existing literature, including research on emotional eating, behavior change, and remote interventions. The discussion reflects on how psychological improvements may support long-term outcomes, and the results are situated within the broader context of service delivery challenges in obesity care.
Discussion	18. Limitations	Limits of generalizability, bias, or precision	✓ The manuscript acknowledges limitations such as the lack of a control group, small sample size, reliance on self-reported weight, and limited generalizability due to sample demographics. These issues are discussed transparently with regard to their impact on validity and interpretation.
Discussion	19. Conclusions	Usefulness and sustainability	✓ The study concludes that remotely delivered SWMSs may support NHS services by offering feasible, scalable care for patients with obesity. Early findings support further evaluation and highlight areas for service refinement, such as physical activity support and diverse recruitment.
Other	20. Funding	Sources of funding	✓ The evaluation was

			conducted as part of routine service delivery under an existing NHS contract with Second Nature Healthy Habits Ltd. No external funding was received. Authors' time was covered internally and included contributions to service design, intervention development, data analysis, and manuscript preparation.
Other	21. Conflicts of Interest	Author relationships disclosed	✓ All authors are employees of Second Nature Healthy Habits Ltd, and this is clearly disclosed in the manuscript's "Conflicts of Interest" section.
